# Fine-Tuning of the Cpx Envelope Stress Response Is Required for Cell Wall Homeostasis in *Escherichia coli*

**DOI:** 10.1128/mBio.00047-16

**Published:** 2016-02-23

**Authors:** Antoine Delhaye, Jean-François Collet, Géraldine Laloux

**Affiliations:** a de Duve Institute, Université catholique de Louvain, Brussels, Belgium; b WELBIO, Brussels, Belgium

## Abstract

The envelope of Gram-negative bacteria is an essential compartment that constitutes a protective and permeability barrier between the cell and its environment. The envelope also hosts the cell wall, a mesh-like structure made of peptidoglycan (PG) that determines cell shape and provides osmotic protection. Since the PG must grow and divide in a cell-cycle-synchronized manner, its synthesis and remodeling are tightly regulated. Here, we discovered that PG homeostasis is intimately linked to the levels of activation of the Cpx system, an envelope stress response system traditionally viewed as being involved in protein quality control in the envelope. We first show that Cpx is activated when PG integrity is challenged and that this activation provides protection to cells exposed to antibiotics inhibiting PG synthesis. By rerouting the outer membrane lipoprotein NlpE, a known Cpx activator, to a different envelope subcompartment, we managed to manipulate Cpx activation levels. We found that Cpx overactivation leads to aberrant cellular morphologies, to an increased sensitivity to β-lactams, and to dramatic division and growth defects, consistent with a loss of PG homeostasis. Remarkably, these phenotypes were largely abrogated by the deletion of *ldtD*, a Cpx-induced gene involved in noncanonical PG cross-linkage, suggesting that this transpeptidase is an important link between PG homeostasis and the Cpx system*.* Altogether our data show that fine-tuning of an envelope quality control system constitutes an important layer of regulation of the highly organized cell wall structure.

## IMPORTANCE

The envelope of Gram-negative bacteria is essential for viability. First, it includes the cell wall, a continuous polymer of peptidoglycan (PG) that determines cell morphology and protects against osmotic stress. Moreover, the envelope constitutes a protective barrier between the cell interior and the environment. Therefore, mechanisms called envelope stress response systems (ESRS) exist to monitor and defend envelope integrity against harmful conditions. Cpx is a major ESRS that detects and manages the accumulation of misfolded proteins in the envelope of *Escherichia coli*. We found that this protein quality control system also plays a fundamental role in the regulation of PG assembly. Strikingly, the level of Cpx response is critical, as an excessive activation leads to phenotypes associated with a loss of cell wall integrity. Thus, by contributing to PG homeostasis, the Cpx system lies at the crossroads between key processes of bacterial life, including cell shape, growth, division, and antibiotic resistance.

## INTRODUCTION

The envelope of Gram-negative bacteria is a complex multilayered compartment that is essential for viability and plays a crucial defensive role against various environmental assaults. It constitutes therefore a major target for current antibiotics. The envelope consists of two concentric membranes, the inner membrane (IM) and outer membrane (OM), which are separated by the periplasm, a compartment containing a continuous monolayer of peptidoglycan (PG). The PG, also called the cell wall, is a stiff polymer of glycan chains cross-linked by peptide bridges that determines the shape of bacteria and offers protection against osmotic stress (1).

Bacteria have evolved elaborate quality control strategies to monitor and maintain the integrity of their envelope. In the model bacterium *Escherichia coli*, several envelope stress response systems (ESRS) have been identified. These systems, which span the envelope, detect perturbations and mount a repair or preventive action by controlling the expression of appropriate genes (2–4). One of the primary ESRS is called Cpx, which senses and responds to envelope alterations that were mostly associated with protein misfolding in the periplasm (5, 6). A classical two-component system forms the core machinery of the Cpx pathway: inducing signals trigger a phosphotransfer between the sensor histidine kinase CpxA at the IM and the cytoplasmic response regulator CpxR, which controls the transcription of a vast regulon (7, 8). Additional factors modulate this system, such as the OM lipoprotein NlpE, which induces the Cpx response when overexpressed (9, 10). Of note, the nature of Cpx-inducing cues is strikingly diverse and some signals are not related to envelope processes directly, such as the translation inhibitor gentamicin (11). Nevertheless, the main outputs generated by the activation of Cpx are an increased production of envelope chaperones and proteases and a drop in the synthesis of several bulky and energy-consuming membrane complexes. Thus, Cpx serves as a protein quality control system decreasing the envelope burden under stressful conditions (12). Interestingly, data from a couple of recent studies suggest that the Cpx system may also detect perturbations to the cell wall in *E. coli*. First, the Cpx response is activated by the simultaneous absence of four nonessential penicillin-binding proteins (PBPs), which are PG-modifying enzymes (13). Second, several genes that belong to the Cpx regulon are upregulated in the presence of antibiotics that block steps of PG synthesis, although in this case, the direct role of CpxR remains to be determined (14). Moreover, Cpx was recently shown to control the transcription of genes implicated in PG regulation (15). The assembly of PG is a critical aspect of bacterial life, since it underlies the fundamental processes of cell elongation (for lateral growth) and division (to build the new poles of the progeny). Therefore, PG synthesis and remodeling are tightly regulated in space and time, in part via the assembly of complex multiprotein machineries known as the elongasome and the divisome (1).

Here we set out to investigate and dissect the potential and intriguing connection between the Cpx system and the PG. We show that Cpx, a system traditionally viewed as monitoring protein homeostasis in the cell envelope, is also intimately connected to PG assembly. Importantly, we demonstrate that precise control of the degree of Cpx activation is critical to maintain cell wall integrity and we identify LdtD, a transpeptidase catalyzing noncanonical PG cross-links, as a major connector between PG homeostasis and Cpx. Finally, we show that the Cpx system responds massively to the mislocalization of the lipoprotein NlpE, a property that can be used to tune the levels of Cpx activation.

## RESULTS

### Antibiotics specifically inhibiting elongation or division or inactivating MreB induce the Cpx envelope stress response.

**FIG 1:**
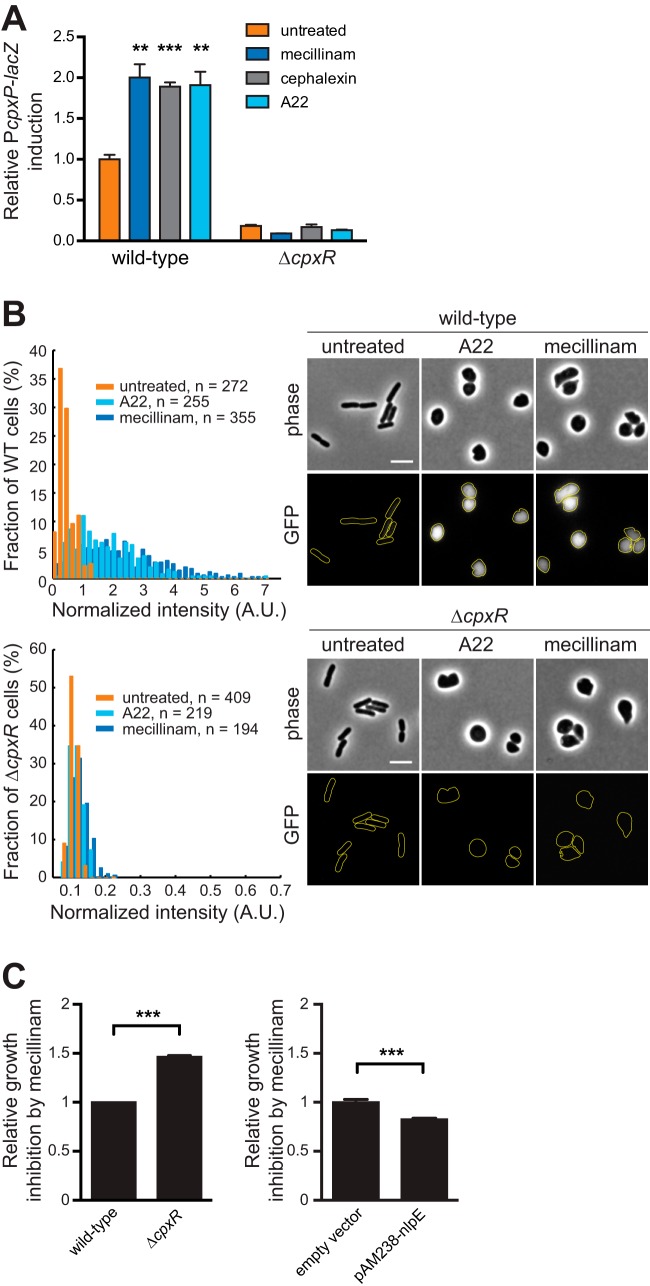
Antibiotics inhibiting essential PG synthesis enzymes or MreB activate the Cpx envelope stress response. (A) β-Lactams and A22 induce the expression of the specific CpxR activity reporter P*cpxP-lacZ.* Wild-type (GL43) and *cpxR*:*:kanR* (GL73) cells were incubated with or without amdinocillin (mecillinam) (0.3 µg/ml), cephalexin (10 µg/ml), or A22 (5 µg/ml) for 1 h before measurement of β-galactosidase activity. A.U., arbitrary units. All values were normalized by the average activity obtained for untreated wild-type cells. Bars represent the average of normalized values from three independent clones. Error bars represent the standard error of the mean (SEM). (B) β-Lactams and A22 induce the expression of the specific Cpx reporter P*cpxP-gfpmut2.* Wild-type (GL368 [top]) and Δ*cpxR* (GL382 [bottom]) cells carrying the P*cpxP-gfpmut2* reporter on a plasmid were incubated with or without amdinocillin or A22 as in panel A before imaging. (Left) distribution of total GFP fluorescence intensity per cell normalized by cell area obtained from cells displayed on the right. (Right) Phase-contrast and GFP fluorescence images of representative cells treated or not with amdinocillin or A22. (C) Relative growth inhibition zone around amdinocillin Sensi-Discs. The diameter of growth inhibition was measured around Sensi-Discs containing 10 µg amdinocillin after overnight incubation. Each value was normalized by the average diameter obtained for the control strain. Bar graphs represent averages ± SEM of normalized values from at least three biological replicates for each strain (GL43 [control] and GL73 on the left, GL63 [control] and GL62 on the right). For all panels, ** indicates *P* ≤ 0.01 and *** indicates *P* ≤ 0.001.

To formally examine if perturbation of PG synthesis induces the Cpx response, we measured the activity of the *cpxP* promoter (P*cpxP*), a reliable reporter of the response regulator CpxR (5, 7), following addition of antibiotics targeting PG assembly. We used concentrations of antibiotics that did not arrest cell growth under the tested conditions, to avoid any secondary toxicity effect. By measuring β-galactosidase activity from a chromosomal P*cpxP-lacZ* fusion, we found that a 1-h treatment with amdinocillin (mecillinam), a β-lactam antibiotic inhibiting the PG transpeptidase PBP2 (which causes cell rounding), increased Cpx activity by about 2-fold (Fig. 1A; see Fig. S1A and S1B in the supplemental material). Similar results were obtained with cephalexin, a β-lactam inhibiting PBP3 and leading to cell filamentation. Thus, the specific inactivation of essential PG synthesis components of the elongasome (PBP2) or divisome (PBP3) activates the Cpx response. The rod shape of *E. coli* is maintained by coupling PG synthesis with the actin-like cytoskeleton element MreB. MreB polymerizes in dynamic short filaments distributed perpendicularly to the long cell axis and closely associates with PG synthases, ensuring uniform PG synthesis along the long axis of the cell (16–19). Interestingly, depolymerization of MreB by addition of subinhibitory concentrations of the drug A22 [*S*-(3,4-dichlorobenzyl)isothiourea], which impacts PG synthesis and makes the cells become round (20–22), also activated the Cpx system (Fig. 1A; see Fig. S1A and S1B). In all of the assays described above, no activity was detected in a *cpxR* deletion strain, showing that induction of the reporter by β-lactams or A22 is fully dependent on the Cpx system (Fig. 1A). Of note, this effect was independent on the OM lipoprotein NlpE (see Fig. S1C), as it is for most known Cpx-inducing cues (see Discussion). Because measurement of β-galactosidase activity only provides an overview of the Cpx response at the population level, it remained possible that β-lactams and A22 highly induce this pathway in a minority of cells within that population, while most cells remain unaffected. Thus, in a second assay, we fused the gene encoding a fast-folding variant of the green fluorescent protein (GFPmut2) to P*cpxP*—used as single-cell readout for Cpx activity—and quantified the fluorescence intensity in cells imaged after treatment or not with amdinocillin or A22 (Fig. 1B). After 1 h of treatment, cells were round as expected when PBP2 or MreB is inhibited (20, 21, 23). The distribution of the GFPmut2-associated fluorescence signal (normalized by cell area to account for the cell shape variations) clearly shifted toward higher values when cells were grown in the presence of amdinocillin or A22. This increase was also completely abolished in a Δ*cpxR* background (Fig. 1B). Hence, our data clearly demonstrate that the Cpx response is induced upon direct inactivation of the division or elongation machinery by antibiotics targeting essential PBPs or perturbing the spatial organization of PG synthesis.

### Cpx activation protects cells exposed to β-lactams.

Our observations, together with previous data showing that the Cpx two-component system is induced in a strain lacking several nonessential PBPs (13), suggest that the Cpx response provides a repair or protective mechanism against insults to PG homeostasis. Hence, we hypothesized that the activation of the Cpx system could offer a fitness advantage to *E. coli* cells exposed to β-lactams. Indeed, we found that turning Cpx off by a *cpxR* deletion increased *E. coli* sensitivity to amdinocillin and other β-lactams (Fig. 1C; see Fig. S1D in the supplemental mtaterial). This was not due to a compromised permeability function of the OM since Δ*cpxR* cells were not more susceptible than wild-type cells to several antibiotics that cannot readily cross the membrane (see Fig. S1E). On the other hand, Cpx induction by NlpE overexpression rendered cells slightly but significantly more resistant (Fig. 1C; see Fig. S3 in the supplemental material), which was not observed when a Cpx-unrelated OM lipoprotein (RcsF) was overexpressed (see Fig. S1F). Altogether, these data indicate that the Cpx system participates in PG homeostasis by sensing cell wall pertubations and mounting a response to minimize the damage.

### Overactivation of the Cpx system by CpxA mutants leads to growth, division, and shape abnormalities.

**FIG 2:**
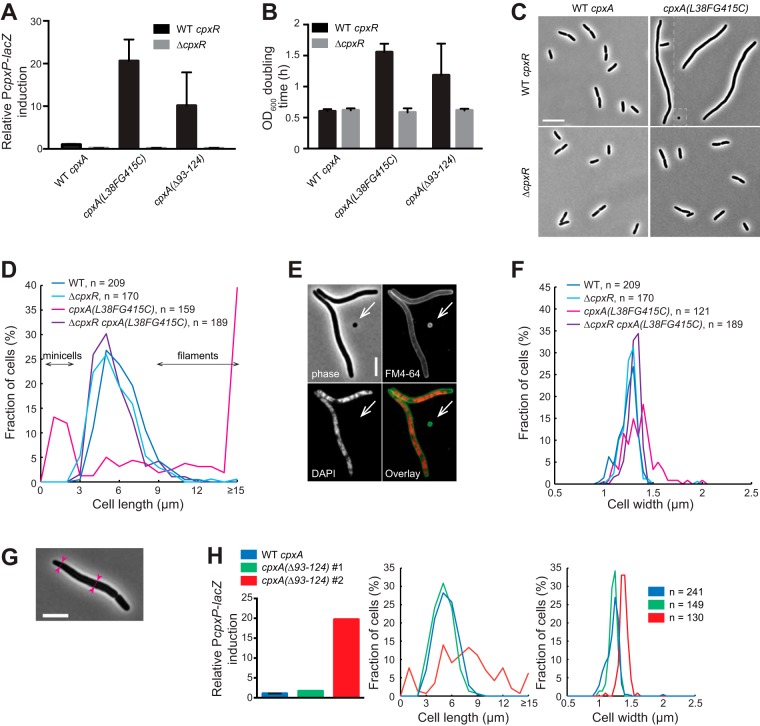
Overactivation of the Cpx system by CpxA* leads to cell growth, division, and shape abnormalities. (A) Cells carrying mutations in *cpxA* (CpxA*) overactivate the Cpx response. P*cpxP-lacZ* reporter activity of cells carrying the wild-type *cpxA* or the *cpxA*_L38F G415C_ or *cpxA*_Δ93–124_ variants (wild-type *cpxR* strains, GL43, GL388, and GL402; Δ*cpxR* strains GL73, GL390, and GL389). All values were normalized by the average activity obtained for untreated wild-type cells (GL43). Bars represent the averages of normalized values from 3 independent clones ± standard deviations (SD). (B) OD_600_ doubling times of CpxA* strains in the presence or absence of *cpxR* (strains as in panel A). Values are averages from 3 independent clones ± SD. (C) Phase-contrast images of representative cells expressing wild-type CpxA or CpxA_L38F G415C_ (strains GL43, GL73, GL388, and GL390). White dashed lines indicate different fields of view juxtaposed here for display purposes. (D) Length distributions for cells imaged in panel C. *n* indicates the number of cells. (E) Minicells are formed in a population of CpxA_L38F G415C_ cells. Cells were labeled with the membrane dye FM4-64 (green on the overlay image) and DAPI to stain DNA (red). Most minicells are devoid of DNA (arrow). Scale bar, 5 µm. (F) Width distributions for cells imaged in panel C considering cells longer than 2 µm to avoid the contribution of minicells. *n* indicates the number of cells. (G) Pink arrowheads joined by dashed lines show regions of different widths in a cell expressing CpxA_L38F G415C_ (GL427, grown at 30°C before phase-contrast imaging). Bar, 5 µm. (H) Relative P*cpxP-lacZ* reporter activity (calculated as in panel A), cell length, and cell width distributions for wild-type *cpxA* cells and two representative colonies of a strain carrying the *cpxA*_Δ93–124_ construct. The color keys for the length and width distributions are the same as for the P*cpxP-lacZ* reporter activity. *n* indicates the number of cells.

Because all processes involved in PG synthesis need to be strictly regulated, we asked if tight control of the level of Cpx response is also required for PG homeostasis. To address this question, we took advantage of previously identified mutations in *cpxA* (cpxA*) that constitutively activate the Cpx response (24). CpxA* variants lack the phosphatase activity exhibited by many sensor kinases of two-component systems (25), which favors the accumulation of the phosphorylated, active form of CpxR in the absence of an inducing signal. We first constructed CpxA* strains by exchanging the *cpxA* open reading frame (ORF) on the chromosome by a cpxA* construct (encoding either CpxA_L38F G415C_ or CpxA_Δ93–124_). Both strains showed 10- to 20-fold increased CpxR-dependent activity on average compared to the wild-type strain (this was higher than that upon induction by NlpE overexpression [see below]) (Fig. 2A). Interestingly, we noticed that both CpxA* strains displayed significant growth defects: mass doubling times were at least twice as long as those of wild-type cells or CpxA* strains in which *cpxR* had been deleted (Fig. 2B). Thus, overactivation of the Cpx response negatively affects cell growth. Of note, we repeatedly observed greater variability of Cpx activation levels and doubling times in the CpxA_Δ93–124_ strain (Fig. 2A and B), which could indicate the occurrence of suppressor mutations. Strikingly, CpxA* strains also displayed morphological aberrations (Fig. 2C), which, together with the observed growth defect, are consistent with a general loss of PG homeostasis. First, most CpxA_L38F G415C_ cells looked elongated or filamentous and DNA-free minicells were observed (Fig. 2C to E), suggesting a division defect and reminiscent of previous observations (see Discussion) (26). Furthermore, additional hallmarks of PG deregulation were observed in CpxA_L38F G415C_ cells, including cell widening (Fig. 2F), irregular cell width (Fig. 2G), and occasional lysis (not shown). Interestingly, all morphological defects were alleviated when the Cpx system was turned off by deleting *cpxR* (Fig. 2C, D and F), strongly suggesting that it is the overactivation of the two-component system that leads to a global loss of PG homeostasis impacting growth, division, and shape. Further supporting this notion, we found that the CpxA_Δ93–124_ clones that highly activated the Cpx response were elongated, whereas the clones with a low Cpx activity (possible suppressors) displayed normal morphological aspects (Fig. 2H). Thus, there was a remarkable correlation between the level of Cpx activation and the extent of morphological aberrations.

### Rerouting the lipoprotein NlpE to the inner membrane also overactivates the Cpx system.

**FIG 3:**
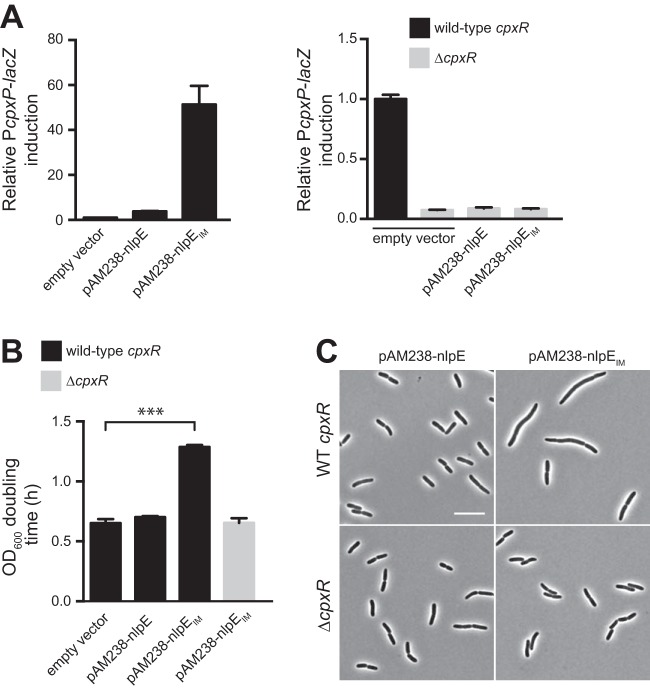
Growth and morphological defects of cells with Cpx overactivation are not restricted to CpxA* mutants. (A) The Cpx system responds massively to mislocalized NlpE. P*cpxP-lacZ* reporter activity of strains expressing NlpE or NlpE_IM_*.* β-Galactosidase activity was measured in wild-type *cpxR* (left) and *cpxR* deletion strains (right) containing an empty pAM238 vector or pAM238 carrying the wild-type *nlpE* or *nlpE*_IM_ (GL63, GL62, or GL99 and GL331, GL136, or GL140, respectively). All values were normalized by the average activity obtained for control cells (GL63). Bars represent the average normalized values from at least 3 independent clones. Error bars indicate SEM. (B) OD_600_ doubling times calculated for cells containing an empty pAM238 vector or pAM238 carrying the wild-type *nlpE* or *nlpE*_IM_ in wild-type *cpxR* or *cpxR* deletion backgrounds as indicated (strains from left to right: GL63, GL62, GL99, and GL140). Bars represent average values from 3 independent clones. Error bars indicate SEM. ***, *P* ≤ 0.001. (C) Phase-contrast images of representative wild-type *cpxR* and *cpxR* deletion cells expressing NlpE or NlpE_IM_ (GL62 or GL99 and GL136 or GL140, respectively). Scale bar, 10 µm.

Even though the growth and morphological defects of CpxA* strains were abolished by deletion of *cpxR*, it remained possible that this PG deregulation phenotype is somehow specific to Cpx induction by the CpxA* variants. To test this, we sought to stimulate a strong Cpx response using an alternative mechanism. Overexpression of the OM lipoprotein NlpE has frequently been used as a tool to activate the Cpx system (9, 10, 12). While the molecular mechanism behind this observation remains mysterious, an attractive hypothesis is that a fraction of the overproduced NlpE fails to be sorted to the OM and accumulates in the IM, where it could directly or indirectly activate CpxA (as suggested in references 9 and 27). We reasoned that if that was the case, rerouting the whole population of NlpE to the IM should induce the Cpx response more than overexpression of the wild-type protein. We modified selected residues of the lipobox of NlpE according to lipoprotein sorting rules (28) in order to change its final destination and produce an IM-anchored lipoprotein (NlpE_IM_), which was verified by membrane fractionation (see Fig. S2A and B in the supplemental material). We first expressed this mislocalized form in an *nlpE* deletion strain to avoid any contribution of the native, properly targeted protein. As predicted, NlpE_IM_ turned on the Cpx system more strongly than the OM-localized NlpE (Fig. 3A). This is in agreement with previous data suggesting that IM-targeted NlpE induces the Cpx response (27), although in that case, the activity of a different, nonspecific Cpx reporter was monitored (29). The effect of mislocalized NlpE was not restricted to strains lacking the native copy of *nlpE*, since similar Cpx activity was observed when the mislocalized NlpE form was produced in a strain carrying the native, chromosomal *nlpE* (data not shown). Furthermore, in these experiments, NlpE_IM_ was produced at comparable levels to the wild-type protein expressed from the same plasmid, excluding the possibility that the strong Cpx response is due to larger amounts of NlpE (see Fig. S2A). Hence, our data show that the Cpx system is especially sensitive to mislocalized NlpE.

### Growth and morphological defects upon Cpx overactivation are not restricted to CpxA* mutants.

Next, we asked if Cpx overactivation upon NlpE mislocalization also affected several PG-related aspects. First, we found that the growth rate of cells expressing the mislocalized variant of NlpE (NlpE_IM_) was slowed down (Fig. 3B), similar to our observations with CpxA* strains (Fig. 2B). This shows that the growth defect occurs as a result of a strong Cpx response, independently of mutations in a core member of the two-component system. Consistently, NlpE_IM_-carrying cells were also characterized by dramatic morphological aberrations, including cell filamentation, minicell formation, and larger cell width, which were completely abolished in the absence of the response regulator CpxR (Fig. 3C; see Fig. S2C and D in the supplemental material). All phenotypes induced by mislocalized NlpE were independent of the presence of the chromosomal copy of *nlpE* (data not shown), ruling out possible effects of either the coexistence of two different forms of the protein or the deletion of the native *nlpE*.

### A member of the Cpx regulon implicated in PG cross-linking contributes to the growth and morphological defects in Cpx overactive cells.

**FIG 4:**
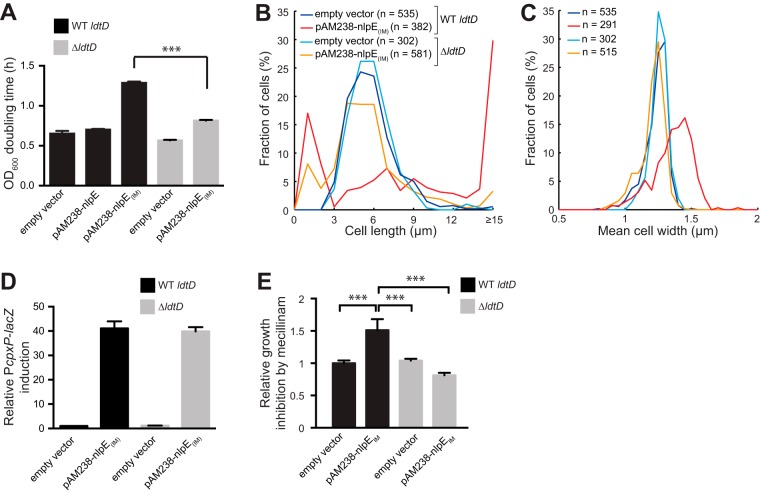
A gene of the Cpx regulon implicated in PG cross-linking contributes to the growth and morphological defects of Cpx overactive cells. (A) OD_600_ doubling times calculated for cells carrying the indicated plasmids in wild-type *ldtD* or *ldtD* deletion backgrounds as shown (strains GL63, GL62, GL99, GL394, and GL396). Bars represent average values from 3 independent clones. Error bars indicate SEM. ***, *P* ≤ 0.001. (B) Length distributions of cells carrying the indicated plasmids in wild-type *ldtD* or *ldtD* deletion backgrounds (GL63, GL99, GL394, and GL396). *n* indicates the number of cells. (C) Width distributions for cells used in panel B considering cells longer than 2 µm to avoid contribution of minicells. Line colors are as in panel B. *n* indicates the number of cells. (D) β-Galactosidase activity from the P*cpxP-lacZ* reporter in cells (wild-type *ldtD* or *ldtD* deletion) carrying the indicated plasmids (strains GL63, GL99, GL394, and GL396). All values were normalized by the average activity obtained for control cells (GL63). Bars represent the average normalized values from at least 3 independent clones. Error bars indicate SEM. (E) Overactivation of Cpx by mislocalized NlpE increases sensitivity to PG perturbation by amdinocillin (mecillinam) in an *ldtD-*dependent manner. The diameter of growth inhibition was measured around Sensi-Discs containing 10 µg amdinocillin after overnight incubation. Each value was normalized by the average diameter obtained for the control strain. Bar graphs represent averages of normalized values from 3 biological replicates for each strain (GL63 [control], GL99, GL394, and GL396). Error bars indicate the SEM. ***, *P* ≤ 0.001.

Taken together, our data show that turning on the Cpx response to high levels (i.e., more than 10-fold compared to basal activity) produces pleiotropic effects on growth, shape and division, likely reflecting misregulation of PG synthesis and/or remodeling. Since CpxR up-regulates at least three genes known to play a role in shaping the PG structure (8, 15, 30, 31), we hypothesized that some of the observed defects could be alleviated by the deletion of one or several of these genes. To test this, we expressed NlpE_IM_ in single-deletion strains lacking either *ldtD* (also known as *ycbB*), *slt*, or *ygaU*, three of the identified PG modifier genes controlled by Cpx (8, 15). Remarkably, the absence of *ldtD*, but not of *slt* or *ygaU* (data not shown), significantly improved cell growth (Fig. 4A), reduced the amount of filaments (Fig. 4B), and restored normal cell width (Fig. 4C), without preventing Cpx overactivation by the IM-targeted NlpE variant (Fig. 4D). Moreover, mislocalization of NlpE (high Cpx response) rendered wild-type cells more sensitive to amdinocillin (Fig. 4E) and other β-lactams (see Fig. S3 in the supplemental material), and deletion of *ldtD* suppressed this sensitivity (Fig. 4E). Thus, cells with high levels of Cpx activation display growth and morphological defects and become sensitized to antibiotics targeting PG synthesis in a manner that depends, at least partially, on *ldtD*.

## DISCUSSION

### The Cpx system senses PG damage.

The simultaneous absence of four nonessential PBPs turns on the Cpx response, supporting an extended role of this system in monitoring perturbations in the cell wall (13). We now provide compelling evidence for this by showing that Cpx senses inhibition of essential components of both the elongasome and the divisome, as well as the loss of spatial coordination of PG synthesis during growth. However, the molecular nature of the signal that stimulates the Cpx two-component system when cells are grown in the presence of PG-perturbing compounds like β-lactams or the MreB inhibitor A22 remains to be identified. Cpx likely senses PG damage independently of NlpE since the absence of this lipoprotein did not prevent induction of the Cpx response by β-lactams or A22 (see Fig. S1C in the supplemental material), consistent with the fact that NlpE is dispensable for Cpx activation by most inducing cues (5). Actually, it is still unresolved how the kinase activity of CpxA is triggered by the accumulation of misfolded proteins in the envelope, which is considered a major Cpx-activating signal. Clearly, further investigation is needed to shed light on how a protein quality control system such as Cpx is activated by cell wall alterations and other envelope stresses.

### Cpx overactivation leads to division, growth, and shape defects that are largely dependent on *ldtD.*

A previous study had reported division defects in CpxA* strains, but it was unknown if this phenotype was due to a strong induction of the Cpx response or to side effects of the CpxA* mutations (26). Here we demonstrate that a robust Cpx response triggered either by CpxA* or NlpE mislocalization gives rise to several problems pointing to a general imbalance in PG assembly and remodeling. Moreover, these phenotypes were largely suppressed by deletion of the CpxR-induced gene *ldtD*, identifying LdtD as a major functional link between the observed PG-related defects and Cpx. Deletion of either *slt* or *ygaU*, two other Cpx-induced genes with a known PG-modulating function (15), did not alleviate the morphological aberrations, while double *ldtD slt* or *ldtD ygaU* deletions did not improve the compensatory effect of *ldtD* deletion alone (data not shown), consistent with the primary role of *ldtD*. However, overexpression of *ldtD* by itself was not sufficient to recapitulate the morphological and growth defects generated by Cpx overactivation (data not shown). Thus, one or more unidentified Cpx-dependent factors may be needed to produce these LdtD-dependent phenotypes. Interesting to note, exponentially growing cells lacking *ldtD* did not trigger the Cpx response (Fig. 4D), contrary to results obtained by Bernal-Cabas et al. using the same reporter (15). The fact that these authors used cells growing in the late exponential phase, a condition known to induce the Cpx system (5), could explain this discrepancy.

Recently, mutations in *cpxA* were shown to suppress the lethality caused by the loss of the PG amidase AmiB in *Pseudomonas aeruginosa*, suggesting a role for Cpx in PG-related aspects of cell division in this bacterium (32). Interestingly, these alleles did not confer growth defects, which could reflect species-specific differences in the strengths of the Cpx responses generated by these CpxA variants or in the connection between Cpx activation levels and PG regulation.

### How does LdtD contribute to PG-linked defects in division, growth, and shape?

LdtD is an l,d-transpeptidase that generates normally poorly abundant cross-links between diaminopimelic acid (DAP) residues (31, 33). Consistently, a constitutive, moderate Cpx activation (~10-fold higher than the basal level) increases the overall degree of cross-linkage of the sacculus, partly by increasing the relative amount of DAP-DAP bonds (15, 24). In this case, the cell wall is further stabilized (33). Our results suggest that in strains with a disproportionate Cpx response, structural modifications of the PG and the abundance of DAP-DAP cross-links in particular cause defects in cell division, growth, and morphology instead of strengthening the sacculus.

Since the sequential process of cell division is intimately coupled to the assembly and remodeling of the PG structure, a severe imbalance in the different types of bonds in the PG mesh by upregulation of LdtD, and possibly by other Cpx-dependent effects, may have direct disruptive consequences on divisome assembly, stability, or constriction. For instance, DAP-DAP-bridged muropeptides are poor substrates for amidases (34), yet the action of amidases at the septum contributes to the formation of denuded glycan chains that help recruiting the late divisome component FtsN (35), a protein that favors stabilization and constriction of the cytokinetic FtsZ ring driving cell septation (36, 37). Thus, an attractive hypothesis to link the observed division defects with LdtD overexpression is that large amounts of DAP-DAP bonds weaken the recruitment of FtsN, leading to a less stable and/or efficient cytokinetic ring. This idea is consistent with the filamentation phenotype of Cpx overactive cells and the mitigation of this effect in an *ldtD-*deficient strain. Moreover, it is supported by our observations that only a few CpxA* cells that initiated constriction displayed a clear band of GFPmut2-tagged FtsZ (see Fig. S4A in the supplemental material), while most filamentous CpxA* cells displayed dynamic FtsZ clusters or bands (see Fig. S4B), indicating an unstable divisome. We verified that this phenotype was not due to lower FtsZ protein levels (see Fig. S4C).

In addition, we propose that the overproduction of LdtD also impairs cell growth and shape: by causing a strong increase in overall cross-linkage and of DAP-DAP bonds in particular, it could hinder both the incorporation of PG precursors into the preexisting mesh and the release of old material. In another scenario, an excess of LdtD could generate an imbalanced enzymatic composition of the large multiprotein complexes involved in PG remodeling, thereby affecting growth and shape.

### The degree of Cpx response determines sensitivity to a loss of PG integrity.

Our data show that sensitivity to β-lactam is affected by the extent of Cpx response. In this context, we propose the following model. As Cpx is turned on up to moderate levels (similar to those obtained when overexpressing NlpE), the tolerance of cells to a loss of PG integrity increases because of a gradual strengthening of the cell wall thanks to Cpx-induced cross-linkage and DAP-DAP bonds. Indeed, the synthesis of l,d-peptide bonds like DAP-DAP is insensitive to β-lactams and an increased content of this type of cross-links in the PG has been associated with increased resistance to these antibiotics (38). However, when a threshold level of Cpx activation is reached, LdtD (and possibly additional PG-modifying enzymes) is produced in excess. This generates structural modifications to the PG sacculus, including large amounts of DAP-DAP, such that cells are sensitized to additional PG insults.

Remarkably, Cpx plays a critical role in the virulence of bacterial pathogens (39). For several species, the degree of Cpx response changes during the process of infection and is critical for the survival of bacteria inside and outside their host (40–42), further emphasizing the importance to keep this broad-spectrum stress response system in check.

### Conclusions.

**FIG 5:**
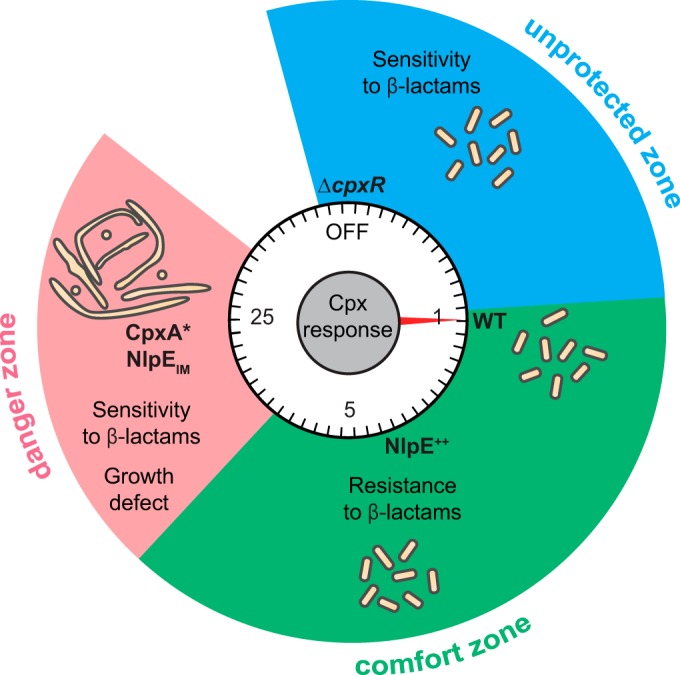
Fine-tuning of the Cpx envelope stress response is critical for cell wall homeostasis. Mild activation of Cpx system by overexpressed NlpE (NlpE^++^) improves resistance to β-lactam antibiotics, while cells unable to activate the system (“OFF” [Δ*cpxR*]) are more sensitive than wild-type cells with basal Cpx response (WT), defining an “unprotected zone” and a “comfort zone” based on the degree of Cpx activation. However, excessive activation of the Cpx system by mislocalized NlpE (NlpE_IM_) or by CpxA* mutants gives rise to a loss of PG homeostasis, manifested by cell division and shape defects, slower growth, and increased sensitivity to β-lactams (“danger zone”). Cell morphology and the induction level of the Cpx response relative to the wild type are represented schematically.

Our major findings are recapitulated in Fig. 5. First, our study challenges the traditional view of the Cpx envelope stress response as a protein quality control system, by providing clear evidence for a connection between Cpx and PG homeostasis. Moreover, we show that fine-tuning the Cpx stress response is critical to maintain cell wall homeostasis, affecting cell growth, morphology, division, and antibiotic resistance. Our study also identifies *ldtD*, a Cpx-induced gene involved in noncanonical PG cross-bridging, as a functional link between PG remodeling and Cpx. Finally, we show that modifying the localization of the lipoprotein NlpE within the envelope can be used to modify the levels of the Cpx response.

## MATERIALS AND METHODS

### Bacterial strains, media, and plasmids.

All strains and plasmids, including construction methods, can be found in Table S1 in the supplemental material. Primers are listed in Table S2 in the supplemental material. Cells were grown in LB medium at 37°C, except when indicated. For strains carrying cpxA* mutations, starter overnight cultures were always grown at 30°C to minimize the occurrence of suppressors as suggested in reference 26. To avoid any effect of Cpx activation that starts in the late exponential phase (5, 43), all experiments were performed from cultures grown until the early or mid-log phase (optical density at 600 nm [OD_600_] of ≤0.6) after diluting an overnight inoculum usually 1:1,000 (or never less than 1:500) in order to ensure exit from the stationary phase. Antibiotics were used for plasmid maintenance when appropriate at the following concentrations: ampicillin, 200 µg/ml; spectinomycin, 50 µg/ml; kanamycin, 50 µg/ml; and chloramphenicol, 20 µg/ml.

### β-Galactosidase assays.

β-Galactosidase activity was assayed as described previously (44). Bar graphs were prepared using Prism 6 (GraphPad Software, Inc.) or Microsoft Excel.

### Antibiotic sensitivity assay.

To measure sensitivity to β-lactams, Sensi-Discs were prepared on the day of the assay by adding a 20-µl drop containing 10 µg amdinocillin, 10 µg ampicillin, or 30 µg cephalexin dissolved in water on 6-mm paper discs (Becton Dickinson). To measure susceptibility to other antibiotics (see Fig. S1E in the supplemental material), we used 6-mm paper discs preloaded with 30 µg vancomycin, 5 µg novobiocin, 10 µg bacitracin, 15 µg erythromycin, or 25 µg rifampin (Becton Dickinson). Cells at an OD_600_ of 0.5 (100 µl or 1 ml) were mixed with LB top agar (3 or 10 ml), containing spectinomycin when required for the maintenance of pAM238 vectors, and poured on top of an LB agar plate. Sensi-Discs were placed on the solidified mixture at a minimum 3-cm distance from each other and from the plate border. The assay was performed in triplicate for each strain. The diameter of growth inhibition around each disc was measured after overnight incubation at 37°C. When indicated, the relative growth inhibition was calculated as follows: each diameter value was normalized by the average diameter obtained from three replicates of the control strain, and the resulting triplicates of normalized values were then averaged for each tested strain. Bar graphs were prepared using Prism 6 (GraphPad Software, Inc.).

### Microscopy image acquisition and analysis.

Live cells were spotted on 1% agarose pads (prepared with phosphate-buffered saline [PBS]) between a glass slide and a coverslip. When indicated, cells were stained with 5 µg/ml DAPI (4′,6-diamidino-2-phenylindole) (Sigma-Aldrich) and 5 µg/ml FM4-64 (Life Technologies) just before imaging. Image acquisition, analysis, and processing were performed as described previously (45) using filter sets 31, 49, and 46 (Carl Zeiss) to image FM4-64, DAPI, and GFPmut2-associated fluorescence, respectively. We used a parameter set modified from algorithm 1 of the MicrobeTracker suite (46) to obtain subpixel cell outlines. Quantitative analysis and plots from the MicrobeTracker data were done on MATLAB (MathWorks, Inc.) using homemade scripts. The values of cell width were obtained by dividing the cell area by the cell length.

### Growth analysis.

Single colonies were used to inoculate overnight cultures, which were diluted 1:500 in round-bottom 96-well plates in 200 µl LB medium with antibiotics when appropriate for plasmid maintenance. Absorbance measurement was performed at 600 nm every 5 min in a Synergy H1 microplate reader (BioTek) with constant orbital shaking at 37°C. Doubling time, *t*, was calculated as *t* = log_2_ /*k*, *k* being the growth rate calculated as the maximum slope from 10 consecutive points on the semilogarithmic curves. Bar graphs were prepared using Prism 6 (GraphPad Software, Inc.)

### Membrane fractionation.

Overnight cultures were diluted 1:500 and grown at 37°C. For each strain, 400 ml of cells was harvested when the OD_600_ reached 0.6 and cell fractionation was performed using a two-step sucrose gradient as described previously in order to separate the IM from the OM (45). ImageJ (http://imagej.nih.gov/ij) was used to quantify the percentage of NlpE detected in each fraction.

### Western blotting.

Proteins from exponentially growing cultures were precipitated with trichloroacetic acid as described previously (47), solubilized in 1× nonreducing Laemmli buffer (48) (with the volume for each sample adapted to normalize all samples by OD_600_), and boiled before being loaded on precast NuPAGE Bis-Tris gels (Life Technologies). Western blotting was performed using standard procedures with the following primary antibodies: anti-FtsZ (rabbit polyclonal serum; AgriSera), anti-PtsI, anti-NlpE, anti-Lpp (rabbit sera; CER Group, Marloie, Belgium), or anti-DsbDα (49) followed by a horseradish peroxidase (HRP)-conjugated anti-rabbit antibody (Sigma). Chemiluminescence signal was imaged with a GE ImageQuant LAS4000 camera (GE Healthcare Life Sciences).

### Statistical analysis.

Statistical tests were performed using Prism 6 (GraphPad Software, Inc.). For Fig. 1A and Fig. S1B in the supplemental material, we used an unpaired one-tailed Student’s *t* test with assumed equal variances (with each condition compared to the untreated control). For Fig. 1C and 4A and Fig. S1C, we used an unpaired two-tailed Student’s *t* test with assumed equal variances for compared groups. For Fig. 3B and 4E and Fig. S1F, we used an unpaired, one-way analysis of variance (ANOVA) with Sidak’s multiple comparison test, with a single pooled variance. For Fig. S1A, S1D, and S1E and Fig. S3 in the supplemental material, we used a two-way ANOVA with Sidak’s multiple comparison test. The reported *P* values for all ANOVA tests were adjusted for multiplicity. For all statistical tests, *P* values of <0.05 were considered statistically significant.

### Figure preparation.

All figures were prepared using Adobe Illustrator CS6 (Adobe Systems, Inc.).

## SUPPLEMENTAL MATERIAL

Figure S1  The Cpx system responds to PG-perturbing antibiotics. (A) Increased activity from the P*cpxP-lacZ* reporter is detected before 1 h of treatment with β-lactams or A22*.* Wild-type (GL43) cells were incubated with or without amdinocillin (mecillinam) (0.3 µg/ml), cephalexin (10 µg/ml), or A22 (5 µg/ml), and β-galactosidase activity was measured at the indicated times after addition of the drug. For each time point, all values were normalized by the average activity obtained for untreated cells (dashed line, value of 1). Bars represent the average of normalized values from three independent clones. (B) Cpx activation by lower concentrations of PG-perturbing antibiotics. β-Galactosidase activity was measured from wild-type cells (GL43) treated for 1 h with antibiotics at various concentrations as indicated. Note that none of the A22 or amdinocillin treatments induced cell lysis; only a few lysed cells were observed with 10 µg/ml cephalexin and not with lower concentrations of this drug. All values were normalized by the average activity obtained for untreated cells (dashed line, value of 1). Bars represent the average of normalized values from independent clones. (C) NlpE is not required for Cpx activation by PG-perturbing antibiotics. Wild-type (WT [GL43]) and Δ*nlpE* (GL44) cells were incubated with or without amdinocillin (0.3 µg/ml), cephalexin (10 µg/ml), or A22 (5 µg/ml), and β-galactosidase activity was measured 1 h after addition of the drug. All values were normalized by the average activity obtained for untreated wild-type cells. Bars represent the average of normalized values from at least three independent clones treated with the indicated antibiotics. (D) Cpx-deficient cells are more sensitive to β-lactams than wild-type cells. The relative growth inhibition zone around Sensi-Discs containing 10 µg amdinocillin, 10 µg ampicillin, or 30 µg cephalexin was obtained as in Fig. 1C. Bar graphs represent averages of normalized values from at least three biological replicates for each strain (GL43 [WT] and GL73 [Δ*cpxR*]). (E) Cpx-deficient cells are not more permeable to antibiotics than wild-type cells. The diameter of growth inhibition was measured around Sensi-Discs containing antibiotics that poorly cross the OM, except if its permeability function is compromised, because of their hydrophobicity (novobiocin, bacitracin, erythromycin, and rifampin) or their large size (vancomycin). Bar graphs represent averages from three independent clones for each strain: GL43 (WT), GL73 (Δ*cpxR*), and CB47 (Δ*surA*, used as positive control for compromised OM permeability function) (50). (F) Relative growth inhibition around amdinocillin Sensi-Discs was calculated as for Fig. 1C for strains GL62 (pAM238-nlpE), GL63 (pAM238) (empty vector used as a control for normalization), and GL245 (pAM238-rcsF). Bar graphs represent averages from 6 replicates. For all panels, error bars indicate the SEM. ***, *P* < 0.001; **, *P* < 0.01; *, *P* < 0.05; ns, not significant. Download Figure S1, EPS file, 1.4 MB

Figure S2  Overactivation of Cpx by mislocalized NlpE leads to morphological aberrations. (A) NlpE_IM_ and wild-type NlpE are overproduced at similar levels. The Western blot shows protein levels of NlpE expressed from its native chromosomal locus (wild-type *nlpE*, strain GL43) and NlpE or NlpE_IM_ expressed from the pAM238 vector (wild-type *nlpE*, strain GL62; *nlpE*_IM_, strain GL99), as indicated. (B) NlpE_IM_ mostly localizes at the IM. Cell lysates (GL43, native WT NlpE; GL62, WT NlpE expressed from pAM238; GL99, NlpE_IM_ expressed from pAM238) were subjected to a two-step sucrose gradient to collect the membrane fraction and separate the OM from the IM. Final fractions were analyzed by Western blotting using antibodies raised against NlpE, Lpp (control for OM localization), and DsbD (IM control). Graphs show the percentage of total NlpE that was detected in each analyzed fraction. Bands of Lpp and DsbD detected by Western blotting and the subcellular localizations are shown below the corresponding fractions for each strain. Data are from a representative experiment out of two repeats. White lines indicate when distant lanes from the same membrane have been placed next to each other for display. Note that none of the overexpressed variants is fully confined to one membrane; this is likely due to overexpression since this localization “leakiness” was less observed when NlpE was produced at native levels. Nevertheless, these data confirm that we could indeed modify the subcellular distribution of NlpE as expected from the lipoprotein sorting rules. (C) Length distribution of cells imaged in Fig. 3C and wild-type *cpxR* cells carrying the empty pAM238 vector as indicated. *n* indicates the number of cells. (D) Width distributions of wild-type or *cpxR* deletion strains expressing NlpE_IM_, considering cells imaged in Fig. 3C that were longer than 2 µm to avoid the contribution of minicells. The lines’ color key is the same as in panel B. *n* indicates the number of cells. Download Figure S2, TIF file, 2 MB

Figure S3  Overactivation of Cpx sensitizes cells to several β-lactams. Growth inhibition was measured and presented as in Fig. 1C and 4E for strains GL63 (pAM238) (empty vector used as control), GL62 (pAM238-nlpE), and GL99 (pAM238-nlpE_IM_). Sensi-Discs contained 10 µg amdinocillin (mecillinam), 10 µg ampicillin, or 30 µg cephalexin. ***, *P* ≤ 0.001. Download Figure S3, EPS file, 0.9 MB

Figure S4  Localization of FtsZ in CpxA* cells indicates divisome instability. (A) Localization of GFPmut2-FtsZ in a strain expressing CpxA_L38FG415C_. Cells (GL427) carrying a low-copy-number plasmid for the expression of GFPmut2-FtsZ were grown at 30°C before imaging. Shown are phase-contrast and GFP fluorescence images of a constricting cell (same as in Fig. 2G). The arrow points at the GFPmut2-FtsZ ring at the constriction site. Bar, 5 µm. (B) Time-lapse image of a representative cell grown as in panel A showing the dynamic localization pattern of GFPmut2-FtsZ (phase-contrast image at time zero) and fluorescence signal from GFPmut2-FtsZ over time (in minutes) for the indicated cell region. White arrowheads point at occasional band-like patterns; yellow arrowheads point at dynamic clusters. (C) Western blot showing similar FtsZ protein amounts in a wild-type strain (lane 1, GL43) and a CpxA* strain (lane 2, GL388). The cytosolic PtsI protein was detected in parallel as a loading control. The white line between lanes indicates that lanes from the same blot were juxtaposed for display. Download Figure S4, EPS file, 2.3 MB

Table S1  Strains and plasmids used in this study. Included are relevant genotypes and features, construction methods, and sources. Table S1, DOCX file, 0.1 MB

Table S2  Primers used in this study. Included are primer names and sequences. Table S2, DOCX file, 0.1 MB
