# Supplementary figures and images for: Fine-Tuning of the Cpx Envelope Stress Response Is Required for Cell Wall Homeostasis in Escherichia coli
Source: mBio. 2016 Feb 23;7(1):e00047-16. doi: 10.1128/mBio.00047-16 (PMC4791840; doi:10.1128/mBio.00047-16)

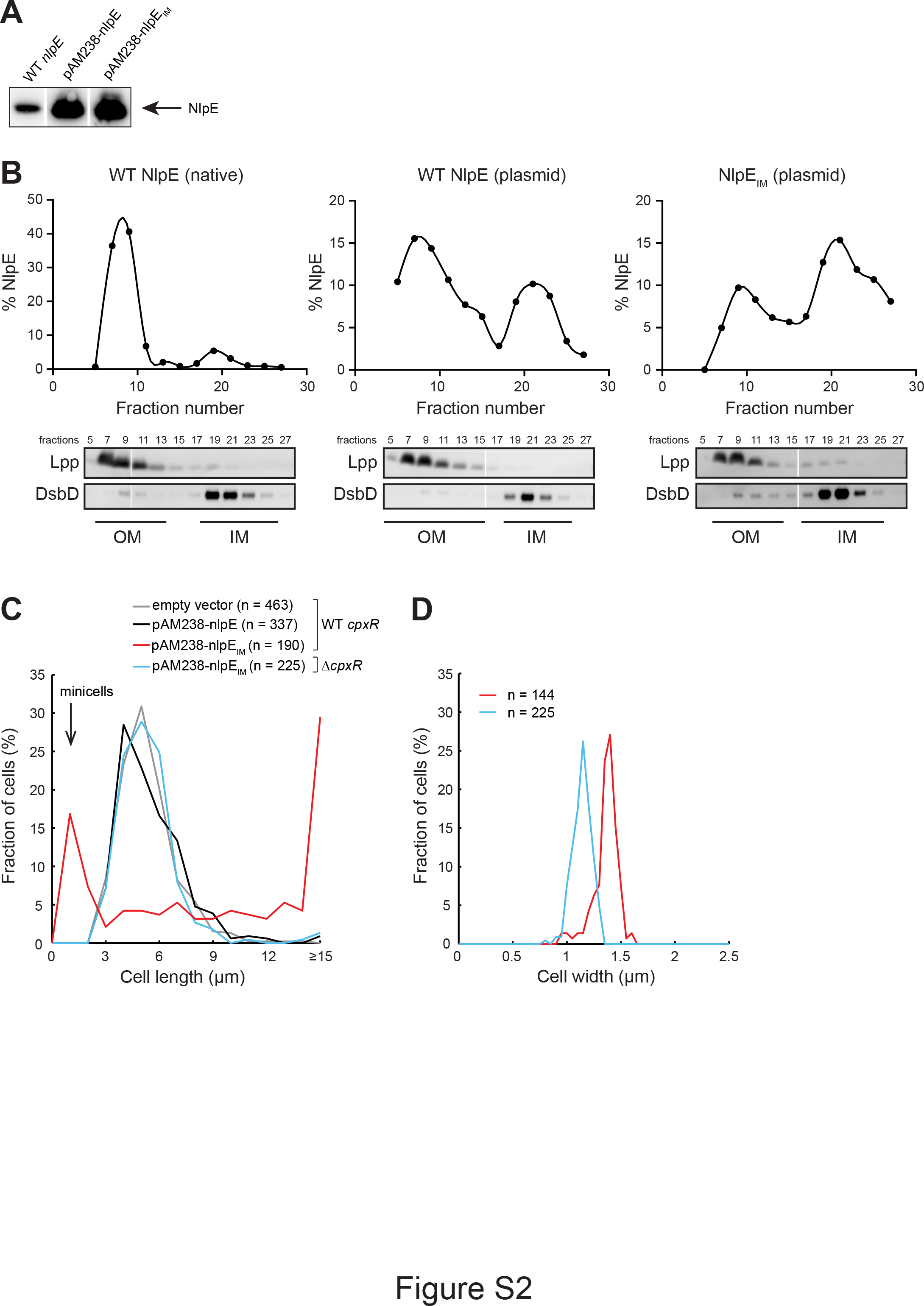

Supplement: Figure S2 — Overactivation of Cpx by mislocalized NlpE leads to morphological aberrations. (A) NlpEIM and wild-type NlpE are overproduced at similar levels. The Western blot shows protein levels of NlpE expressed from its native chromosomal locus (wild-type nlpE, strain GL43) and NlpE or NlpEIM expressed from the pAM238 vector (wild-type nlpE, strain GL62; nlpEIM, strain GL99), as indicated. (B) NlpEIM mostly localizes at the IM. Cell lysates (GL43, native WT NlpE; GL62, WT NlpE expressed from pAM238; GL99, NlpEIM expressed from pAM238) were subjected to a two-step sucrose gradient to collect the membrane fraction and separate the OM from the IM. Final fractions were analyzed by Western blotting using antibodies raised against NlpE, Lpp (control for OM localization), and DsbD (IM control). Graphs show the percentage of total NlpE that was detected in each analyzed fraction. Bands of Lpp and DsbD detected by Western blotting and the subcellular localizations are shown below the corresponding fractions for each strain. Data are from a representative experiment out of two repeats. White lines indicate when distant lanes from the same membrane have been placed next to each other for display. Note that none of the overexpressed variants is fully confined to one membrane; this is likely due to overexpression since this localization “leakiness” was less observed when NlpE was produced at native levels. Nevertheless, these data confirm that we could indeed modify the subcellular distribution of NlpE as expected from the lipoprotein sorting rules. (C) Length distribution of cells imaged in Fig. 3C and wild-type cpxR cells carrying the empty pAM238 vector as indicated. n indicates the number of cells. (D) Width distributions of wild-type or cpxR deletion strains expressing NlpEIM, considering cells imaged in Fig. 3C that were longer than 2 µm to avoid the contribution of minicells. The lines’ color key is the same as in panel B. n indicates the number of cells. Download [file mbo001162698sf2.tif]
