# Supplementary material for: Fine-Tuning of the Cpx Envelope Stress Response Is Required for Cell Wall Homeostasis in Escherichia coli
Source: mBio. 2016 Feb 23;7(1):e00047-16. doi: 10.1128/mBio.00047-16 (PMC4791840; doi:10.1128/mBio.00047-16)
Supplement: Table S1 — Strains and plasmids used in this study. Included are relevant genotypes and features, construction methods, and sources. [file mbo001162698st1.docx]

**TABLE S1. Strains and plasmids used in this study**

| **Strain** | **Relevant genotype** | **Construction method** | **Source** |
| --- | --- | --- | --- |
| GL15 | MC4100 (wild-type *E. coli* strain) |  | Lab collection |
| GL43 = PAD282 | MC4100 λRS88 [*cpxP′-lacZ*^+^] |  | (1) |
| GL44 = PL447 | MC4100 λRS88 [*cpxP′-lacZ*^+^] *nlpE*::*kanR* | P1 transduction of *nlpE*::*kanR* from the Keio collection (2) into GL43 | Pauline Leverrier |
| GL62 | MC4100 λRS88 [*cpxP′-lacZ*^+^] *nlpE*::*kanR* / pAM238-nlpE | Transformation of GL44 with pAM238-nlpE | This study |
| GL63 | MC4100 λRS88 [*cpxP′-lacZ*^+^] *nlpE*::*kanR* / pAM238 | Transformation of GL44 with pAM238 | This study |
| GL68 | MC4100 *cpxR*::*kanR* | P1 transduction of *cpxR*::*kanR* from the Keio collection (2) into GL15 | This study |
| GL73 | MC4100 λRS88 [*cpxP′-lacZ*^+^] *cpxR*::*kanR* | P1 transduction of *cpxR*::*kanR* from the Keio collection (2) into GL43 | This study |
| GL99 | MC4100 λRS88 [*cpxP′-lacZ*^+^] *nlpE*::*kanR* /  pAM238-nlpE(N22D) | Transformation of GL44 with pAM238-nlpE(N22D) | This study |
| GL101 | MC4100 λRS88 [*cpxP′-lacZ*^+^] / pSIM5-Tet | Transformation of GL43 with pSIM5-Tet | This study |
| GL136 | MC4100 λRS88 [*cpxP′-lacZ*^+^] *cpxR*::*kanR* / pAM238-nlpE | Transformation of GL73 with pAM238-nlpE | This study |
| GL140 | MC4100 λRS88 [*cpxP′-lacZ*^+^] *cpxR*::*kanR* / pAM238-nlpE(N22D) | Transformation of GL73 with pAM238-nlpE(N22D) | This study |
| GL245 | MC4100 λRS88 [*cpxP′-lacZ*^+^] / pAM238-rcsF | Transformation of GL43 with pAM238-rcsF (pSC202, (3)) | This study |
| GL260 | MC4100 λRS88 [*cpxP′-lacZ*^+^] / pGP025 | Transformation of GL43 with pGP025 | This study |
| GL271 | MC4100 λRS88 [*cpxP′-lacZ*^+^] *ldtD*::*kanR* | P1 transduction of *ldtD*::*kanR* from the Keio collection (2) into GL43 (*ldtD* = *ycbB*) | This study |
| GL331 | MC4100 λRS88 [*cpxP′-lacZ*^+^] *cpxR*::*kanR* / pAM238 | Transformation of GL73 with pAM238 | This study |
| GL368 | MC4100 / pGL368 | Transformation of GL15 with pGL368 | This study |

| GL378 | MC4100 λRS88 [*cpxP′-lacZ*^+^] ∆*cpxR* | Transformation of GL73 with pCP20 to flip out the FRT-*kanR*-FRT cassette | This study |
| --- | --- | --- | --- |
| GL382 | MC4100 ∆*cpxR* / pGL368 | Tranformation of GL386 with pGL368 | This study |
| GL386 | MC4100 ∆*cpxR* | Transformation of GL68 with pCP20 to flip out the FRT-*kanR*-FRT cassette | This study |
| GL388 | MC4100 λRS88 [*cpxP′-lacZ*^+^] *cpxA*::*cpxA(L38FG415C)-kanR* | λRed recombineering (4) of *cpxA(L38FG415C)-*FRT-*kanR*-FRT^a^ at the *cpxA* locus in GL101. | This study |
| GL389 | MC4100 λRS88 [*cpxP′-lacZ*^+^] ∆*cpxR cpxA*::*cpxA(∆93-124)-kanR* | λRed recombineering (4) of *cpxA(∆93-124)-* FRT-*kanR*-FRT^a^ at the *cpxA* locus in GL426 | This study |
| GL390 | MC4100 λRS88 [*cpxP′-lacZ*^+^] ∆*cpxR cpxA*::*cpxA(L38FG415C)-kanR* | λRed recombineering (4) of *cpxA(L38FG415C)-* FRT-*kanR*-FRT^a^ at the *cpxA* locus in GL426 | This study |
| GL394 | MC4100 λRS88 [*cpxP′-lacZ*^+^] *ldtD*::*kanR* / pAM238 | Transformation of GL271 with pAM238 | This study |
| GL396 | MC4100 λRS88 [*cpxP′-lacZ*^+^] *ldtD*::*kanR* / pAM238-nlpE(N22D) | Transformation of GL271 with pAM238-nlpE(N22D) | This study |
| GL402 | MC4100 λRS88 [*cpxP′-lacZ*^+^] *cpxA*::*cpxA(∆93-124)-kanR* | λRed recombineering (4) of *cpxA(∆93-124)-* FRT-*kanR*-FRT^a^ at the *cpxA* locus in GL101 | This study |
| GL426 | MC4100 λRS88 [*cpxP′-lacZ*^+^] ∆*cpxR* / pSIM5-Tet | Transformation of GL378 with pSIM5-Tet | This study |
| GL427 | MC4100 λRS88 [*cpxP′-lacZ*^+^] *cpxA*::*cpxA(L38FG415C)-kanR* / pGP025 | Transformation of GL388 with pGP025 | This study |
| CB47 | MC4100 *surA*::*kanR* |  | (5) |
| **Plasmid** | **Features, usage, resistance** | **Construction method** | **Source** |
| pAM238 | IPTG-regulated P*_lac_* (in this study: constitutive expression without inducer), pSC101-based vector, Spec^R^ |  | (6) |
| pAM238-nlpE | Expression of wild-type *nlpE* from pAM238, Spec^R^ | Cloning of *nlpE* ORF and 28 upstream base pairs into pAM238 using KpnI and PstI | Joanna Szewczyk |
| pAM238-nlpE(N22D) | Expression of *nlpE_IM_* from pAM238, Spec^R^ | Site-directed mutagenesis on pAM238-nlpE using primers GL50 and GL51 | This study |
| pBAD18 | Arabinose-inducible P_BAD_, pBR322-based, Amp^R^ |  | (7) |
| pBAD18-cpxA | Used as template for site-directed mutagenesis | Cloning of *cpxA* ORF, amplified from GL15 with primers GL83 and GL84, into pBAD18 using KpnI and HindIII | This study |
| pBAD18-cpxA(L38FG415C) | Used as template for amplification of *cpxA(L38FG415C)* | Site-directed mutagenesis on pBAD18-cpxA using primers GL89 and GL90 | This study |
| pBAD18-cpxA(∆93-124) | Used as template for amplification of *cpxA(∆93-124)* | Site-directed mutagenesis on pBAD18-cpxA using primers GL93 and GL94 | This study |
| pCP20 | FLP^+^, λ *c*I857^+^, λ *_P_*_R_ Rep^ts^, ampicillin, Chlor^R^ (the vector is eliminated after transformation by overnight growth at 37°C) |  | (8) |
| pGL368 | Expression of *gfpmut2* under the control of *PcpxP,* used as fluorescent reporter for CpxR activity, Kan^R^ | *cpxP* promoter region amplified from GL15 using primers GL151 and GL152 and cloned with XhoI and BamHI into a pUA139 backbone, obtained from BamHI and XhoI-digested pUA139-P*ppiA-gfpmut2* (Dharmacon, (9)) | This study |
| pGP025 | Expression of *mut2gfp-ftsZ* from a weakened *trc* promoter; IPTG inducible; Amp^R^ |  | T. den Blaauwen (10) |
| pSIM5-Tet | pSC101 plasmid, *repA*^ts^, *tetRA* (Tet^R^), λ-Red (Gam-Beta-Exo), cI857 (ts λ repressor controlling expression of λ-Red genes) |  | D. Hughes |

^a^ To construct *cpxA(L38FG415C)-*FRT-*kanR*-FRT or *cpxA(∆93-124)-*FRT-*kanR*-FRT used for λRed recombineering (4), *cpxA(L38FG415C)* or *cpxA(∆93-124)* was first amplified with primers GL167 and AD12 from pBAD18-cpxA(L38FG415C) or pBAD18-cpxA(∆93-124). FRT-*kanR*-FRT was then amplified from a GL73 colony with primers AD13 and AD14, and both PCR fragments were finally joined using primers GL167 and AD14 (the G415C mutation in *cpxA(L38FG415C)-*FRT-*kanR*-FRT was found by sequencing after the last PCR).

**REFERENCES**:

1. **DiGiuseppe PA**, **Silhavy TJ**. 2003. Signal detection and target gene induction by the CpxRA two-component system. J Bacteriol **185**:2432–2440.

2. **Baba T**, **Ara T**, **Hasegawa M**, **Takai Y**, **Okumura Y**, **Baba M**, **Datsenko KA**, **Tomita M**, **Wanner BL**, **Mori H**. 2006. Construction of Escherichia coli K-12 in-frame, single-gene knockout mutants: the Keio collection. Mol Syst Biol **2**:2006 0008.

3. **Cho S-H**, **Szewczyk J**, **Pesavento C**, **Zietek M**, **Banzhaf M**, **Roszczenko P**, **Asmar A**, **Laloux G**, **Hov A-K**, **Leverrier P**, **Van der Henst C**, **Vertommen D**, **Typas A**, **Collet J-F**. 2014. Detecting Envelope Stress by Monitoring β-Barrel Assembly. Cell **159**:1652–1664.

4. **Yu D**, **Ellis HM**, **Lee EC**, **Jenkins NA**, **Copeland NG**, **Court DL**. 2000. An efficient recombination system for chromosome engineering in Escherichia coli. Proc Natl Acad Sci USA **97**:5978–5983.

5. **Vertommen D**, **Ruiz N**, **Leverrier P**, **Silhavy TJ**, **Collet J-F**. 2009. Characterization of the role of the Escherichia coli periplasmic chaperone SurA using differential proteomics. Proteomics **9**:2432–2443.

6. **Gil D**, **Bouche JP**. 1991. ColE1-type vectors with fully repressible replication. Gene **105**:17–22.

7. **Guzman LM**, **Belin D**, **Carson MJ**, **Beckwith J**. 1995. Tight regulation, modulation, and high-level expression by vectors containing the arabinose PBAD promoter. J Bacteriol **177**:4121–4130.

8. **Cherepanov PP**, **Wackernagel W**. 1995. Gene disruption in Escherichia coli: TcR and KmR cassettes with the option of Flp-catalyzed excision of the antibiotic-resistance determinant. Gene **158**:9–14.

9. **Zaslaver A**, **Bren A**, **Ronen M**, **Itzkovitz S**, **Kikoin I**, **Shavit S**, **Liebermeister W**, **Surette MG**, **Alon U**. 2006. A comprehensive library of fluorescent transcriptional reporters for Escherichia coli. Nat Methods **3**:623–628.

10. **Ploeger GEJ**. 2009. Ph.D. thesis. University of Amsterdam, Amsterdam, The Netherlands. Functional analysis of ZapA: keeping the one ring together.
