# Supplementary material for: Fine-Tuning of the Cpx Envelope Stress Response Is Required for Cell Wall Homeostasis in Escherichia coli
Source: mBio. 2016 Feb 23;7(1):e00047-16. doi: 10.1128/mBio.00047-16 (PMC4791840; doi:10.1128/mBio.00047-16)
Supplement: Table S2 — Primers used in this study. Included are primer names and sequences. [file mbo001162698st2.docx]

**TABLE S2. Primers used in this study.**

| **Primer** | **5’-3’ sequence** |
| --- | --- |
| AD12 | GGTCGACGGATCCCCGGAATTTAACTCCGCTTATACAGCGG |
| AD13 | CCGCTGTATAAGCGGAGTTAAATTCCGGGGATCCGTCGACC |
| AD14 | CAAATGCCGGATGCGGCGTAAACGCCTTATCCTGCCTGCAAATGCGAAGTTGTAGGCTGGAGCTGCTTCG |
| GL50 | TTACTCTGATGGGATGTGATAATCGGGCCGAAGTCGATAC |
| GL51 | GTATCGACTTCGGCCCGATTATCACATCCCATCAGAGTAA |
| GL83 | AAAAAAGGTACCATGATAGGCAGCTTAACCGC |
| GL84 | AAAAAAAAGCTTTTAACTCCGCTTATACAGCGG |
| GL89 | TCACGCCAGATGACCGAGTTTCTGGATAGCGAACAGCGTC |
| GL90 | GACGCTGTTCGCTATCCAGAAACTCGGTCATCTGGCGTGA |
| GL93 | TATTGGTGACCACCGAAGGCCGCGTGGAACTGGTCGGTCC |
| GL94 | GGACCGACCAGTTCCACGCGGCCTTCGGTGGTCACCAATA |
| GL151 | AAAAAAGGATCCGGGAAGTCAGCTCTCGGTC |
| GL152 | AAAAAACTCGAGCGGCAGCGGTAACTATGCGC |
| GL167 | CCGTGGTTTAAAACCTTGCGTGGTCGCGGCTATCTGATGGTTTCTGCTTCATGATAGGCAGCTTAACCGC |
